# Melatonin and 14-hydroxyed brassinosteroid combined promote kiwifruit seedling growth by improving soil microbial distribution, enzyme activity and nutrients uptake

**DOI:** 10.3389/fpls.2024.1336116

**Published:** 2024-02-08

**Authors:** Xiaoli Zhang, Ting Huang, Yan Liang, Shafiq Hussain, Rui Peng, Tong Wang, Honghong Deng, Jin Wang, Xiulan Lv, Dong Liang, Hui Xia

**Affiliations:** College of Horticulture, Sichuan Agricultural University, Chengdu, China

**Keywords:** kiwifruit, melatonin, 14-hydroxyed brassinosteroid, mineral nutrients, soil microorganisms

## Abstract

Kiwifruit, a nutrient-dense fruit, has become increasingly popular with consumers in recent decades. However, kiwifruit trees are prone to stunted growth after a few years of planting, called early tree decline. In this study, melatonin (MT), pollen polysaccharide (SF), 14-hydroxyed brassinosteroid (14-HBR) were applied alone or in combination to investigate their influence on plant growth, nutrition absorption and rhizosphere bacterial abundance in kiwifruit seedlings. The results revealed that MT, SF and 14-HBR alone treatments significantly increased leaf chlorophyll content, photosynthetic capacity and activities of dismutase and catalase compared with the control. Among them, MT treatment significantly increased the dry root biomass by 35.7%, while MT+14-HBR treatment significant enhanced the dry shoot biomass by 36.9%. Furthermore, both MT and MT+14-HBR treatments markedly improved the activities of invertase, urease, protease and phosphatase in soil, as well as the abundance of Proteobacteria and Acidobacteria in rhizosphere microorganisms based on 16S rDNA sequencing. In addition, MT treatment improved the content of available K and organic matter in soil, and increased the uptake of P, K and Fe by seedlings. In summary, 14-HBR and MT combined had the best effect on promoting rhizosphere bacterial distribution, nutrient absorption and plant growth. These findings may provide valuable guidance for solving growth weakness problem in kiwifruit cultivation.

## 1 Introduction

Kiwifruit (*Actinidia chinensis*) is a deciduous vine fruit tree in *Actinidia* Lindl. of Actinidiaceae family. Over the past century, kiwifruit has been successfully domesticated from wild growth to large-scale cultivation, and the kiwifruit industry has flourished worldwide (Liang et al., 2019). As a nutrient-dense fruit, kiwifruit is rich in vitamin C, dietary fiber, and mineral nutrients and phenolic compounds, *etc.*, thus favored by increasing consumers (Richardson et al., 2018). China, where the fruit originated, has also developed into the world’s largest kiwifruit producer since 2010, and now accounts for more than 60% of the world’s planted area (Ferguson and Huang, 2007). However, in large-scale kiwifruit cultivation, there appeared a decline of tree potential after a few years of planting, called early kiwifruit decline. It was mainly manifested as the cessation of vegetative growth, the decline of fruit yield in the overground, and the loss of fibrous roots or the decay of structural roots in the underground (Bardi, 2020). The possible causes of early kiwifruit decline have been suggested to be associated with root waterlogging, soil anoxic conditions or soil pathogenic microorganisms (Donati et al., 2020). Nevertheless, effective countermeasures for this issue are still lacking.

Plant growth regulators (PGRs) are organic compounds that are synthesized artificially (or extracted from natural organisms) and have similar functions as plant hormones in regulating growth and development (Ahmad et al., 2016; Mohanta et al., 2018). PGRs are widely used in agricultural production to achieve the purpose of increasing yield, improving crop quality and enhancing stress resistance by effectively regulating crops growth process (Spaepen et al., 2014; Kudoyarova et al., 2019). Multiple PGRs have been reported to positively regulate different physiological and biochemical processes in plants under normal and stressed conditions. Melatonin (MT), a newly discovered phytohormone, is involved in diverse plant development process, including root and stem growth, photosynthesis, callus formation, flowering, leaf senescence retardation, reactive oxygen species metabolism, and plant defense system (Jahan et al., 2023; Tan et al., 2014; Zhang et al., 2014). Moreover, MT application enhanced the decomposition of soil organic matter by increasing the activity of soil microbes and enzymes, hence contributing to the improvement of root morphology, root structure, and plant growth (Liang et al., 2018; Li et al., 2018). 14-Hydroxyed brassinosteroid (14-HBR), an important group of plant steroid hormones, performs a crucial role in various physiological functions of plants (Zhang et al., 2013; Ahammed et al., 2020). The application of brassinosteroids enhanced plant growth and biomass accumulation by improving antioxidant enzyme activity under unfavorable environmental conditions (Hussain et al., 2019; Ahammed et al., 2020). Furthermore, it efficiently enhanced the uptake of essential nutrients (N, P, K, Ca, Mg, Fe, Zn, etc.) by improving ion homeostasis in leaves and roots under extreme environmental conditions (Liu et al., 2014; Anwar et al., 2018). Pollen polysaccharide is a novel PGR containing essential nutrients for plant growth and development (Wang et al., 2013). However, little information about its beneficial role in different physiological and biochemical processes occurring in plants is known so far.

Rhizosphere microorganisms, predominantly bacteria, are tightly attached to the rhizosphere soil. The environment influences the composition and abundance of rhizosphere microorganisms, and there are interactions between microorganisms and plant hosts (Zhang et al., 2017). During plant growth, apoptosis roots and secretions serve as crucial sources of nutrition and energy for rhizosphere microorganisms. In comparison to non-rhizosphere soil, rhizosphere soil provides better water status and aeration conditions, thereby creating an optimal microenvironment for the proliferation of these microorganisms. These interactions significantly impact microbial population structure and contribute to the overall health of plant hosts. Numerous microbes in the rhizosphere act as guardians of plant health by protecting plants against pathogens and helping in nutrient acquisition from the soil (Bai et al., 2022).

However, the effect of the above-mentioned PGRs on kiwifruit growth and rhizosphere microorganisms is not well understood so far. In this study, three treatments of single MT, 14-HBR, SF, and two combined treatments MT+SF, 14-HBR+MT were designed to evaluate their effects on plant growth index, soil rhizosphere microbial abundance, and plant uptake of mineral nutrients. We hope the results can lay a foundation for the further wide application of the new PGRs and provide a reference for solving the problem of early kiwifruit decline.

## 2 Materials and methods

### 2.1 Plant preparation and treatment

A pot experiment was conducted in the greenhouse of Sichuan Agricultural University, Chengdu, China. One-year-old kiwifruit (*Actinidia chinensis*) seedlings were planted in pots (30×22 ×32cm), each filled with approximately 10.0 kg orchard soil and placed in a greenhouse with natural sunlight. Soil properties were as fellow: pH 7.08, organic matter 21.38 g/kg, total nitrogen 1.15 g/kg, total phosphorus 1.13 g/kg, total potassium 12.23 g/kg, alkali hydrolysis nitrogen 104.30 mg/kg, available phosphorus 9.87 mg/kg, available potassium 79.03 mg/kg. A total of 144 seedlings, one in each pot, were divided into six groups for treatment. Every treatment contained 15 pots as replicates. The seedlings were treated with: 1) water (CK), 2) 100 µM melatonin solution (MT), 3) pollen polysaccharide diluted 200 times (SF), 4) 14-hydroxyed brassinosteroid diluted 1500 times (14-HBR), 5) 50 µM melatonin + 14-hydroxyed brassinosteroid diluted 1500 times (MT + HBR), and 6) pollen polysaccharide diluted 200 times + 50 µM melatonin (SF+ MT), respectively. The concentration of MT was based to our previous study (Liang et al., 2019), and the concentration of 14-HBR and SF was referred to the manufacturer’s recommended concentration and our preliminary experiments. The treatments were applied three times at 15 days intervals, 200 mL each time. After two months, soil and plant samples were taken for parameters determination. Part of the leaf and soil samples were stored at -80°C for DNA/RNA extraction.

### 2.2 Determination of plant growth parameters

The plant height, stem diameter, root length, and the fresh weight of shoot or root were recorded at 60 d after treatment. The dry weight of shoot and roots were measured after drying to a constant weight at 80°C in an oven.

### 2.3 Determination of root vitality and soil organic matter content

Roots deoxidation capacity was calculated by using the triphenyltetrazolium chloride method (Zhang et al., 2017). Soil organic matter content was estimated following the procedure illustrated by Mylavarapu and Zinati (2009).

### 2.4 Photosynthetic pigment content measurement

The content of chlorophyll a (Chl *a*), chlorophyll b (Chl *b*), total chlorophyll and carotenoid were measured following the procedure described by Liang et al. (2018). Fresh leaf samples (0.5 g) were cut into pieces and immersed in 10 mL of ethanol and acetone (1:1, v/v) solution, and kept under dark conditions for 24 hours. Leaf sap was collected and utilized for the measurement of Chl *a*, Chl *b*, and carotenoid content at 470, 646, and 663 nm, respectively, using a spectrophotometer (Thermo Fisher Scientific, USA).

### 2.5 Assay of gas exchange parameters

On day 10 of the drought treatment, the leaf gas exchange parameters, including net photosynthetic rate (Pn), transpiration rate (Tr), stomatal conductance (Gs), and intercellular CO_2_ concentration (Ci), were measured on fully expanded leaves between 9:00 am to 11:00 am using a portable device LI-COR (Model LI-6400, LI-COR Inc., USA). A red/blue LED light source and a constant flow rate of 500 mL·min^-1^ and CO^2^ concentration of ca. 400 μmol·mol^-1^ under a PAR of 1000 μmol·m^-2^s^-1^ were used.

### 2.6 Determination of antioxidant enzyme activity in leaves

The activities of superoxide dismutase (SOD), peroxidase (POD), and catalase (CAT) were measured following the method of Wang and Huang (2015). 0.2 g of leaf samples were homogenized in a precooled 50 mM phosphate buffer PBS (pH 7.8) containing 1% (w/v) polyvinyl pyrrolidone PVP, 2.0 mM dithiothreitol (DTT) and 0.1 mM ethylene-diaminetetraacetic (EDTA). After centrifugation at 4°C, the supernatant was collected. POD activity was measured at 470 nm using hydrogen peroxide and guaiacol as substrates. SOD activity was estimated by monitoring the phytochemical decline of nitroblue tetrazolium (NBT) at 560 nm. CAT activity determined by recording the decrease in absorbance at 240 nm resulting from the decomposition of hydrogen peroxide (H_2_O_2_).

### 2.7 Gene expression assay

The total RNA was extracted using the Mini RNA Isolation I Kit (Beijing Tianmo Sci & Tech Development Co., Ltd, China) according to the manufacturer’s instructions. The first strand cDNA was obtained by reverse transcribing RNA with PrimeScriptTM RT reagent Kit with gDNA eraser (Perfect Real Time) (Takara, Tokyo, Japan). The gene-specific primers were designed using Primer Premier5 and synthesized by Tsingke Biotechnology Co., Ltd (Beijing, China, **Supplementary Table 1**), *Actin* was used as the reference. Quantitative Real-time PCR was performed on the CFX96 Real-Time System C1000 Thermal Cycler (Bio-RAD, Hercules, CA, USA) using an SYBR^®^ Premix Ex TaqTM II (Tli RnaseH Plus) kit (TaKaRa, Tokyo, Japan). The reaction conditions were as follows: 95 °C for 30 s, followed by 40 cycles of 95 °C for 5 s, and at 52 °C to 55 °C for 30 s. Each sample was subjected to three replicates. The 2^- ΔΔCT^ method was used to calculate the relative mRNA expression level using *Actin* as the reference (Zhou et al., 2022).

### 2.8 Determination of soil enzymes activities

The activity of different soil enzymes was measured according to the methods described by Wang et al. (Wang Y. et al., 2016), and the activity of proteinase was estimated according to the method of Ladd and Butler (Ladd and Butler, 1972). Invertase, urease, proteinase, and phosphatase activities were represented as the amount (mg) of glucose, ammonia-nitrogen, and phenol respectively that was released per gram of dry soil in 24 h.

### 2.9 Estimation of the nutrients content in leaves and soil

Leaf samples were oven-dried at 65°C for two days, weighed, and immediately digested in a sulfuric acid solution. Soil samples were air-dried and ground and sieved (< 1 mm) to determine available nutrient content. The content of alkali nitrogen was determined by the alkaline diffusion method. Available phosphorus was extracted with sodium bicarbonate and determined by Mo-Sb anti-colorimetric method, and the content of available potassium was determined by atomic absorption spectrophotome-try (Greger et al., 2018). The concentrations of Zn, Mn, Cu, and Fe were estimated by ICP-MS (7900 Mass Spectrophotometer, Agilent, Santa Clara, CA, USA). Soil organic matter was determined by the potassium dichromate oxidation method.

### 2.10 Soil bacterial communities analysis based on 16S rDNA sequencing

DNA was extracted from the soil samples (1.0 g) using the E.Z.N.A.^®^Stool DNAKit (D4015, Omega, Inc., USA) according to the manufacturer’s instructions. The region of V3-V4, V4, and V4-V5 of 16S rDNA was amplified. The PCR products were purified by AMPure XT beads (Beckman Coulter Genomics, Danvers, MA, USA) and quantified by Qubit (Invitrogen, Carlsbad, CA, USA). The amplicon pools were prepared for sequencing and the size and quantity of the amplicon library were assessed on Agilent 2100 Bioanalyzer (Agilent, USA) and with the Library Quantification Kit for Illumina (Kapa Biosciences, Woburn, MA, USA), respectively. The libraries were sequenced on the NovaSeq PE250 platform.

Samples were sequenced on an Illumina NovaSeq platform according to the manufacturer’s recommendations, provided by LC-Bio. Paired-end reads were assigned to samples based on their unique barcode and truncated by cutting off the bar-code and primer sequence. Paired-end reads were merged using FLASH. Quality fil-tering on the raw reads was performed under specific filtering conditions to obtain high-quality clean tags according to the fqtrim (v0.94) (https://ccb.jhu.edu/software/fqtrim/index.shtml). After quality-filtering, the obtained sequences were clustered operational taxonomic units (OTUs) at a similarity of 97% using Usearch (version 7.0 http://drive5.com/usearch/). Then feature sequences was normalized using the relative abundance of each sample according to SILVA (release 132, http://www.arb-silva.de) classifier at a confidence threshold of 70%. Alpha diversity and beta diversity were calculated by QIIME2 (https://qiime2.org/), the stacked bar chart were drawn by the R package (v3.5.2). The blast was used for sequence alignment, and the feature sequences were annotated with the SILVA database for each representative sequence (Li et al., 2018).

### 2.11 Statistical analysis

Data were presented as mean ± standard deviation (SD). One way ANOVA was adopted for statistical analysis using SPSS 20.0 software. Tukey’s multiple range test was used to compare the differences between treatments at *P <*0.05 level.

## 3 Results

### 3.1 Effect of PGRs on morphological parameters

All treatments increased the plant growth parameters, including plant height, stem diameter, root length and root vitality compared with CK (**Table 1**). Among the single treatments, MT showed the most obvious effect on promoting root length by 25.2%. The combined treatment of MT and 14-hydroxyed brassinosteroid (14-HBR) further improved the growth promotion of aboveground parts, with plant height and stem diameter increased by 9.1% and 5.6% compared with MT, respectively. Consistent with the growth parameters, each PGR treatment increased the accumulation of fresh and dry weight in both roots and shoots of seedlings. MT treatment had the most significant effect on root dry mass, increasing by 35.7%, while MT+14-HBR treatment had the most significant effect on shoot dry mass accumulation, increasing by 37.0% (**Table 1**).

**Table 1:** Effect of PGRs application on morphological parameters and biomass accumulation in kiwifruit seedlings.

Treatment	Plant height (cm)	Stem diameter (mm)	Root length (cm)	Root vitality (µg/g·h)	Biomass			
					Shoot (g FW)	Root (g FW)	Shoot (g DW)	Root (g DW)
CK	79.0± 1.8 b	4.3± 0.2 b	10.7± 0.9 b	35.2± 3.2 c	172.5± 5.1 b	77.3± 1.1 c	33.6± 2.9 b	14.1± 1.0 b
MT	101.1± 9.9 a	5.3± 0.3 a	13.4± 1.3 a	84.0± 7.2 a	216.4± 9.3 a	96.6± 1.0 a	45.2 ± 2.1 a	19.1± 2.4 a
SF	92.0± 8.8 ab	4.8± 0.2 ab	11.2± 0.9 b	47.7± 3.7 bc	196.1± 5.4 ab	86.0± 4.8 b	40.0 ± 2.4 ab	16.7± 2.2 ab
14-HBR	98.2± 7.6 ab	4.8± 0.3 ab	11.5± 0.5 b	54.0± 4.6 bc	190.0± 14.8 ab	86.6± 0.9 b	37.0 ± 7.9 ab	16.8± 1.9 ab
SF+MT	98.2± 1.4 ab	5.2± 0.5 ab	11.6± 0.6 b	61.5± 5.4 b	208.4± 11.5 a	91.4± 3.3 ab	40.4 ± 1.1 ab	17.7± 0.8 ab
MT+14-HBR	110.3± 2.3 a	5.6± 0.3 a	12.6± 1.2 ab	92.9± 8.7 a	218.4± 1.9 a	95.5± 1.5 a	46.0 ± 1.8 a	19.0± 0.8 a

Values are the mean ± SD (n=5). CK: H_2_O; MT: 100 µM melatonin; SF: 200× pollen polysaccharide; 14-HBR: 1500× 14-hydroxyed brassinosteroid; SF+MT: 200× SF + 50 µM MT; MT+14-HBR: 50 µM MT + 1500×14-HBR. Different letters indicate significant differences (P<0.05) between treatments estimated by Tukey’s multiple range test.

### 3.2 Effect of PGRs on photosynthetic pigments and gas exchange parameters

**Figure 1:**
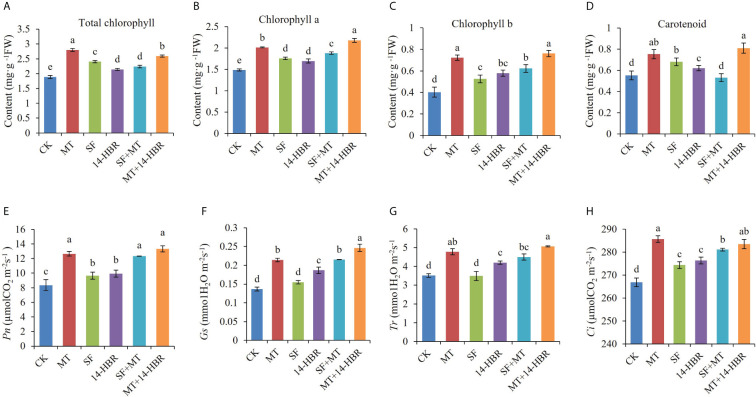
Effect of PGRs application on photosynthetic pigments **(A-D)** and gas exchange parameters **(E-H)** in kiwifruit leaves. Pn: net photosynthetic rate; Gs: stomatal conductance; Tr: transpiration rate; Ci, intercellular CO_2_ concentration. CK: H_2_O; MT: 100 µM melatonin; SF: 200× pollen polysaccharide; 14-HBR: 1500× 14-hydroxyed brassinosteroid; SF+MT: 200× SF + 50 µM MT; MT+14-HBR: 50 µM MT + 1500×14-HBR. Different letters indicate significant differences (*P*<0.05) between treatments estimated by Tukey’s multiple range test.

Our results revealed that all treatments significantly improved the content of photosynthetic pigments in leaves. MT enhanced the content of Chl *a*, Chl *b*, total chlorophyll, and carotenoids by 35.81%, 80%, 48.93%, or 36.36%, respectively (**Figures 1A-D**). For the gas exchange parameter Pn, Tr, Gs, and Ci, treatments MT, SF+MT and MT+14-HBR all had good improvement effects. MT+14-HBR had the best effect, Pn, Gs, Tr and Ci were increased by 59.44%, 84.61%, 44.15% and 4.92% compared with the control, respectively (**Figures 1E-H**).

### 3.3 Effect of PGRs on antioxidant enzyme activity and expression of related genes

**Figure 2:**
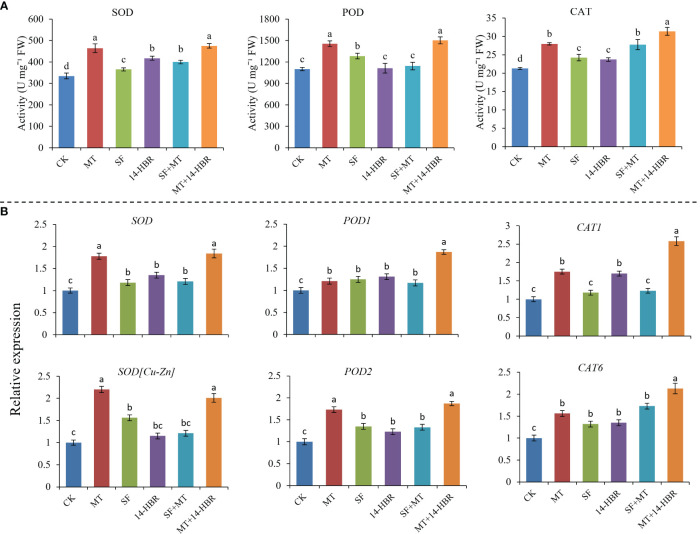
Effect of PGRs application on antioxidant enzyme activities **(A)** and gene expression **(B)**. CAT: catalase, POD: peroxidase, SOD: superoxide dismutase. CK: H_2_O; MT: 100 µM melatonin; SF: 200× pollen polysaccharide; 14-HBR: 1500× 14-hydroxyed brassinosteroid; SF+MT: 200× SF + 50 µM MT; MT+14-HBR: 50 µM MT + 1500×14-HBR. Different letters indicate significant differences (*P*<0.05) between treatments estimated by Tukey’s multiple range test.

All PGR treatments improved the antioxidant activities of CAT and SOD, but 14-HBR and SF+MT did not increase the enzyme activity of POD. MT and MT+14-HBR had the most significant effect, increasing SOD, POD and CAT activities by 42.02%, 36.52% and 47.32%, respectively (**Figure 2A**). Moreover, the expression level of related genes encoding these antioxidants were investigated by qRT-PCR. The expression level of *SOD*, *SOD[Cu-Zn]*, *POD2*, *CAT1* and *CAT6* in MT and MT+14-HBR treatments were significantly up-regulated compared with the control (**Figure 2B**).

### 3.4 Effect of PGRs on soil enzyme activities

**Figure 3:**
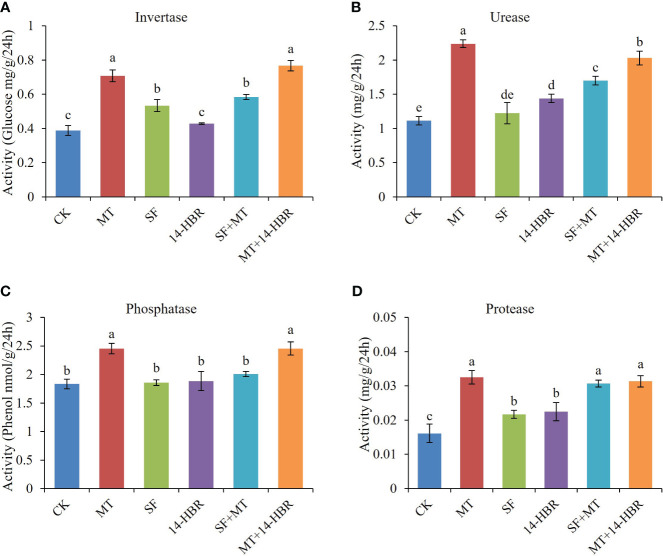
Effect of PGRs application on soil enzyme activity of invertase **(A)**, urease **(B)**, protease **(C)**, and phosphatase **(D)**. CK: H_2_O; MT: 100 µM melatonin; SF: 200× pollen polysaccharide; 14-HBR: 1500× 14-hydroxyed brassinosteroid; SF+MT: 200× SF + 50 µM MT; MT+14-HBR: 50 µM MT + 1500×14-HBR. Different letters indicate significant differences (*P*<0.05) between treatments estimated by Tukey’s multiple range test.

Soil enzymes are associated with nutrient cycling and play a vital role in maintaining soil fertility. The highest values for soil invertase, urease, protease, and phosphatase activity were recorded in MT and MT+14-HBR treatments (**Figure 3**). MT significantly increased the activity of invertase, urease, protease, and phosphatase by 84.21%, 97.66%, 98.75%, and 33.87%, respectively. In addition, MT+14-HBR treatment also enhanced the activity of the above-mentioned enzymes.

### 3.5 Effect of PGRs on mineral nutrients in the soil

**Figure 4:**
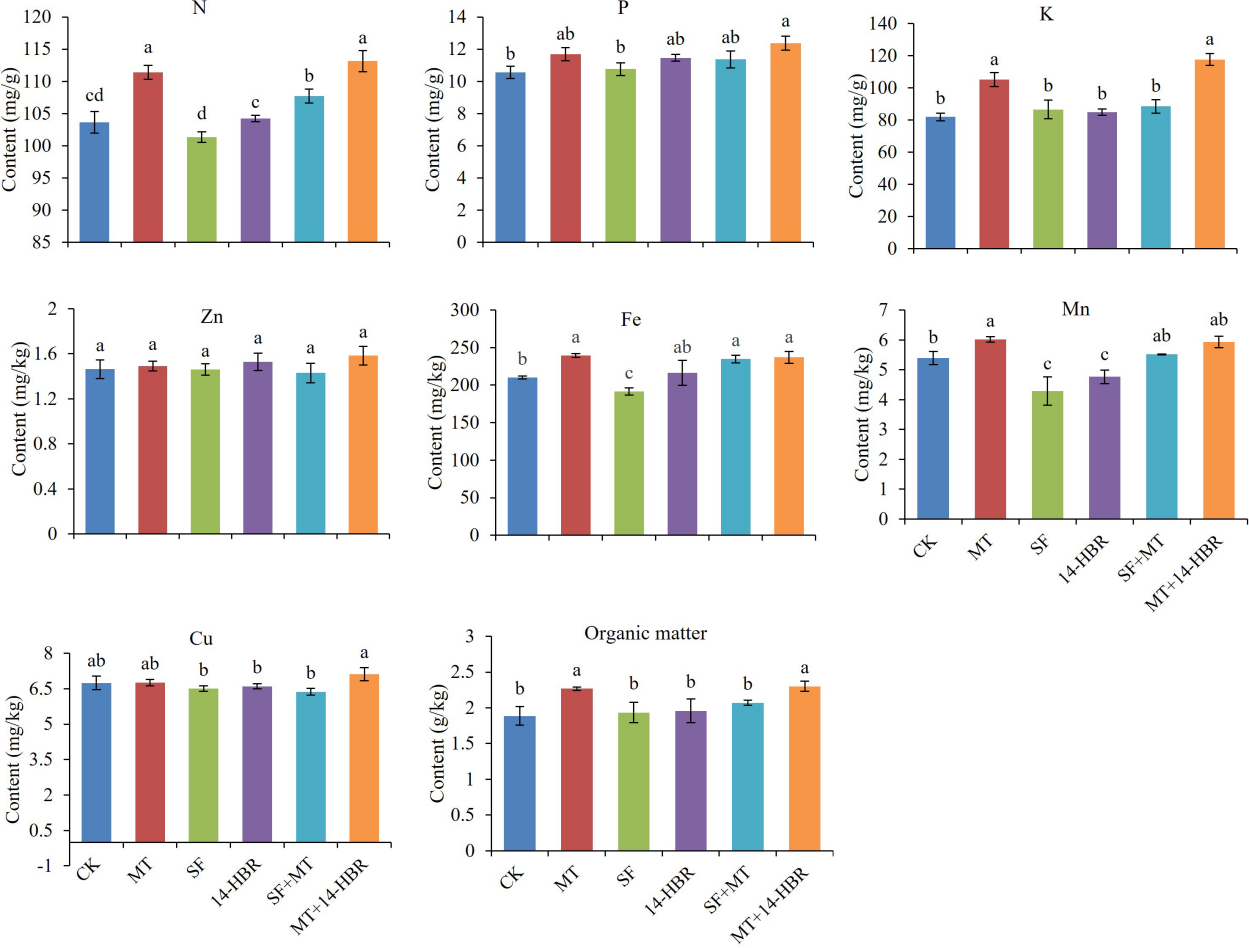
Effect of PGRs application on the content of available nitrogen (N), potassium (K), phosphorus (P), zinc (Zn), iron (Fe), manganese (Mn), copper (Cu), and organic matter in the soil. CK: H_2_O; MT: 100 µM melatonin; SF: 200× pollen polysaccharide; 14-HBR: 1500× 14-hydroxyed brassinosteroid; SF+MT: 200× SF + 50 µM MT; MT+14-HBR: 50 µM MT + 1500×14-HBR. Different letters indicate significant differences (*P*<0.05) between treatments estimated by Tukey’s multiple range test.

Different PGRs treatments significantly increased soil nutrient contents as shown in **Figure 4**. The maximum values for soil nitrogen (N), phosphorus (P), potassium (K), zinc (Zn), copper (Cu), and organic matter content were observed in MT+14-HBR. The MT+14-HBR significantly increased the N, P, K, and organic matter content by 10.48%, 17.36%, 43.54%, or 22.34% than CK. In addition, the highest values for soil Fe and Mn were recorded in MT. The MT significantly enhanced the soil Fe by 13.94% and Mn by 11.50% compared with CK.

### 3.6 Effect of PGRs on microorganism diversity in soil

**Figure 5:**
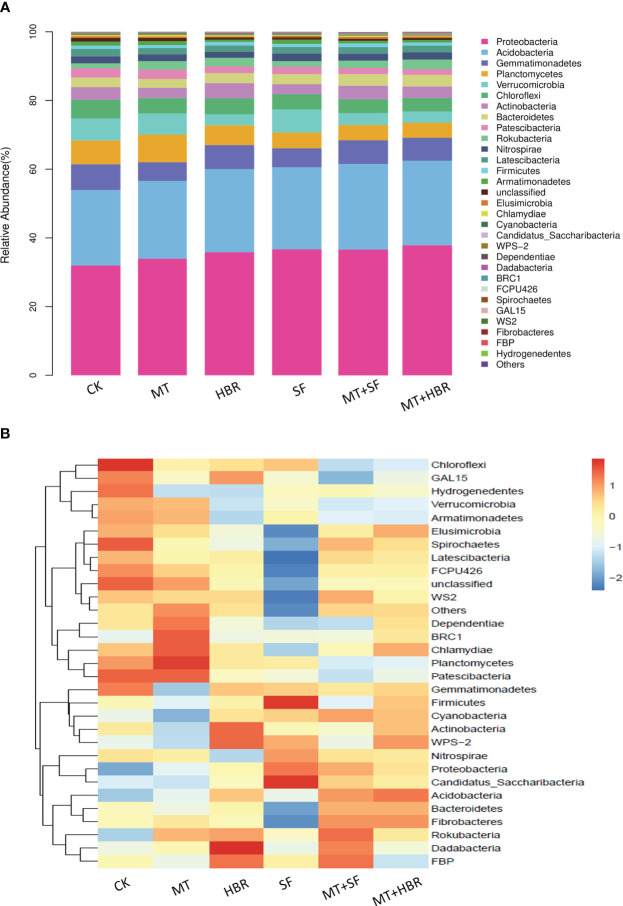
Stacked bar chart **(A)** and heatmap **(B)** of bacterial community diversity in treated soil samples at the phylum level. CK: H_2_O; MT: 100 µM melatonin; SF: 200× pollen polysaccharide; 14-HBR: 1500× 14-hydroxyed brassinosteroid; SF+MT: 200× SF + 50 µM MT; MT+HBR: 50 µM MT + 1500×14-HBR.

All nutrients absorbed by plants must pass through the roots, and the structure of rhizosphere microorganisms affects the uptake of soil nutrients by the roots. Rhizosphere bacterial diversity was therefore analyzed based on 16S rDNA sequencing. 2697-3012 operational taxonomic units (OTUs) were detected in each treated soil sample, with the coverage larger than 0.96, indicating that the depths of sequencing could meet the requirements (**Supplementary Table 2**). A total of 31 phyla of rhizosphere bacteria were detected from soil samples, Proteobacteria, Acidobacteria, Gemmatimonadetes, Planctomycetes, Verrucomicrobia, and Chloroflexi were the dominant bacterial communities in all treatment soil samples, accounting for 80.21%. However, there were differences in the abundance of bacterial communities among treatments. Compared with the control, all PGR treatments enhanced the relative abundance of Proteobacteria and Acidobacteria, and the highest abundance was recorded in MT+14-HBR at 38.92% and 24.96%, respectively (**Figure 5A**). Bacterial communities with significant abundance differences between treatments were analyzed. Chloroflexi, Spirochaetes had the highest abundance in CK; Planctomycetes, Chasmydiae and BRC1 had the highest abundance in MT treatment; Dadabacteria and Actinobacteria had the highest abundance in HBR treatment; Firmicures and Candidatus_Saccharibacteria had the highest abundance in SF treatment; While MT+HBR treatment had more bacterial communities with moderate abundance (**Figure 5B**).

**Figure 6:**
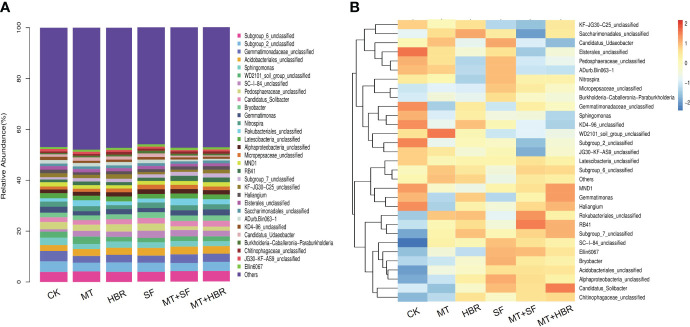
Stacked bar chart **(A)** and heatmap **(B)** of bacterial community diversity in treated soil samples at the genus level. CK: H_2_O; MT: 100 µM melatonin; SF: 200× pollen polysaccharide; 14-HBR: 1500× 14-hydroxyed brassinosteroid; SF+MT: 200× SF + 50 µM MT; MT+14-HBR: 50 µM MT + 1500×14-HBR.

At the genus level, the dominant communities in all soil samples included Subgroup 6, Subgroup 2, Gemmatimonadaceae, Acidobacteriales, and Sphingomonas (**Figure 6A**). The bacterial composition among different soil samples was generally the same, but the proportions of each genus were fluctuation (**Supplementary Figure 1**, **Supplementary Table 3**). For instance, JG36-GS-52 was found only in MT and MT+14-HBR treatments. Lamia and Sphingobacteriaceae were detected in all soil treatments except SF and control treatments. Among all soil treatments, only the SF treatment soil did not show an abundance of Prosthecobacter and Luteolibacter bacterial groups. Soil samples in MT and MT+14-HBR treatments showed the highest abundance of Proteobacteria, Myxococcales, Desulfarculaceae Bdellovibrio, Subgroup 22, Bryobacter, Rokubacteriales, and Anaerolineae, respectively. All PGR treatments significantly increased the abundance of Bradyrhizobium, Alphaproteo-bacteria, Dongia, bacteriap-25, Acidobacteriaceae, and Gemmatirosa compared with the control (**Figure 6B**, **Supplementary Figure 1**).

### 3.7 Effect of PGRs on the absorption of nutrients

**Figure 7:**
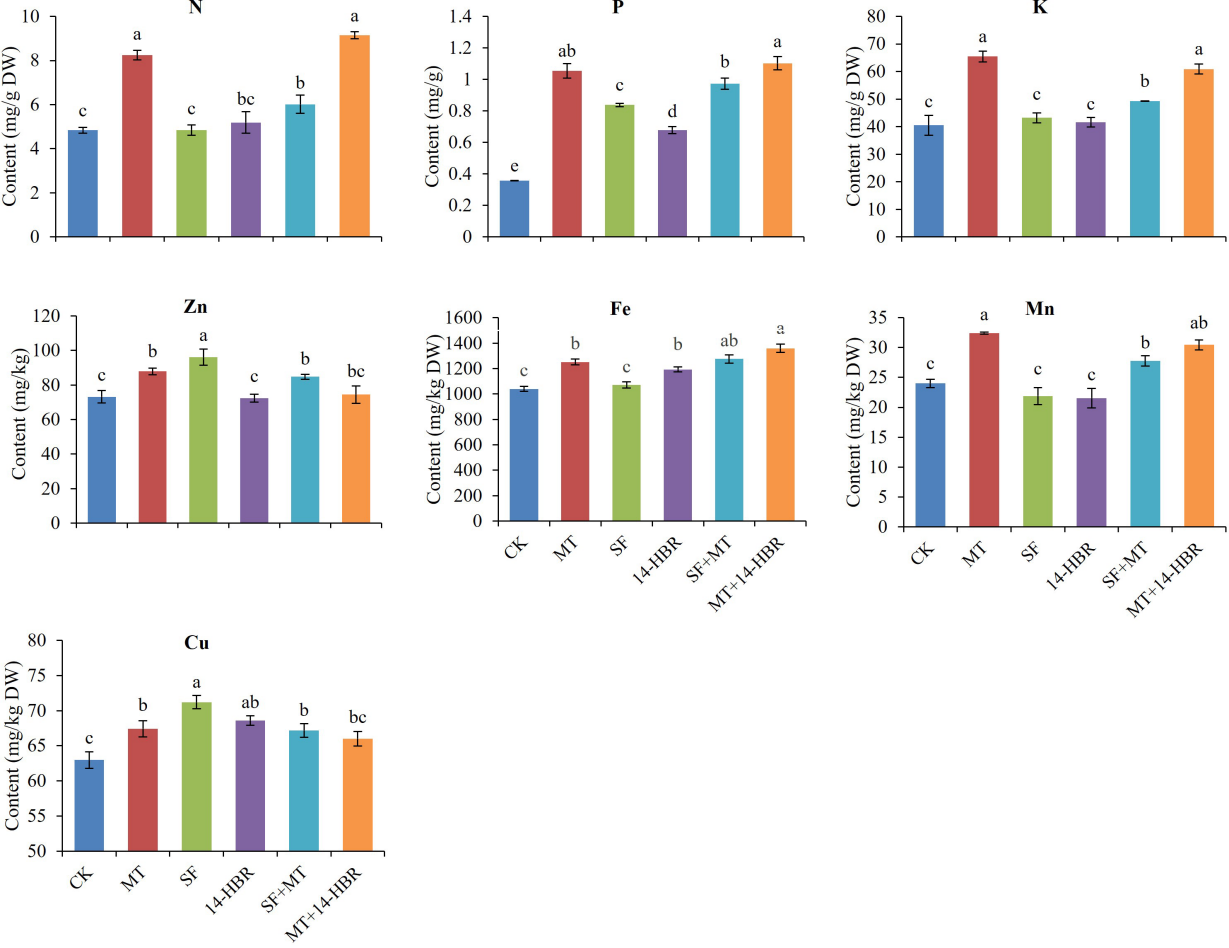
Effect of PGRs application on the content of nutrients in leaves of kiwifruit seedlings. CK: H_2_O; MT: 100 µM melatonin; SF: 200× pollen polysaccharide; 14-HBR: 1500× 14-hydroxyed brassinosteroid; SF+MT: 200× SF + 50 µM MT; MT+14-HBR: 50 µM MT + 1500×14-HBR. Different letters indicate significant differences (*P*<0.05) between treatments estimated by Tukey’s multiple range test.

PGRs application improved nutrient contents of N, P, K, Zn, Fe, Mn and Cu in seedling leaves (**Figure 7**). MT and MT+14-HBR treatments significantly improved the contents of N, P, K, Fe, Mn in leaves, and SF had the best effect to increase the content of Zn and Cu than other treatments. Our findings related to leaf nutrient contents revealed that MT among alone treatments and MT+14-HBR in combined treatments showed the most promising results with a beneficial effect on multiple nutrients.

## 4 Discussion

In this study, the effect of three PGRs, melatonin (MT), pollen polysaccharide (SF), 14-hydroxyed brassinosteroid (14-HBR) on plant growth, nutrient uptake and rhizosphere microbiota distribution in kiwifruit seedlings were investigated, since they play a crucial role in plant development (Chmur and Bajguz, 2021; Sun et al., 2021). We expected to find an effective way to promote plant growth and provide a reference for overcoming the early tree decline in large-scale kiwifruit cultivation, which was reported mainly due to root damage (Donati et al., 2020).

Overall, our results showed that MT, SF and 14-HBR all can increase chlorophyll content, photosynthesis capacity, and promote the kiwifruit seedling growth with an increase of shoot height, stem diameter, root length, and vitality and biomass of root and shoot, as reported in previous studies about the role of melatonin and brassinosteroid (Vardhini and Anjum, 2015; Wei et al., 2015). However, there are few reports about the application of pollen polysaccharide and 14-HBR on plant growth to date. 14-HBR is a natural brassinosteroid product derived from rape pollen that is more active than the other four chemically synthesized brassinolide compounds, including 24-epibrassinolide, 22, 23, 24-epibrassinolide, 28-epibrassinolide and 28-homobrassinolide. Pollen polysaccharide are extracted from the pollen of different plants and is rich in amino acids, proteins, vitamins, flavonoids and other beneficial ingredients (provided by the producer). Herein, our results showed that SF had a comparable effect with 14-HBR on promoting plant growth. Like other PGRs, they increased chlorophyll content, the pigment responsible for photosynthesis to capture and transmit solar energy in plants (Arnao and Hernández-Ruiz, 2009), enhanced net photosynthetic rate to provide more C-source to synthesis for the plant, thus to promoter accumulation of biomass in the plant (Szafrańska et al., 2016; Wang L. et al., 2016; Cui et al., 2017). Encouragingly, melatonin had a stronger effect than SF and 14-HBR almost in all growth parameters. To some extent, it supported melatonin as a master regulator in plants as suggested by Arnao and Hernández-Ruiz (2019).

Since soil environmental microecology is proposed as the main cause for early vine decline in kiwifruit (Donati et al., 2020), we paid special attention to the effects of PGR on soil enzyme activities and rhizosphere microorganisms. Our results showed an improvement in enzyme activity of invertase, urease and phosphatase induced by the addition of all three PGRs, with the treatments MT and MT+14-HBR most, followed by SF+MT. Melatonin have much stronger effect on enhancing urease activity than other PRGs, it may dependent on concentration in certain range, resulting a better effect of 100 μm MT than 50μm MT and 14-HBR (**Figure 3**). Improving soil enzyme activity can provide more nutrients such as sugar and glucose, carbon dioxide and ammonia, inorganic phosphorus to plants and rhizosphere microorganisms, making the soil more healthy (Gianfreda et al., 2005; Krajewska, 2009; Mikhailouskaya and Bogdevitch, 2009).

Soil microorganisms perform important functions in the environment via involvement in various biological as well as physio-chemical reactions occurring in soil, thus directly contributing toward soil fertility and ecosystem stability (Critter et al., 2004). In the present study, we detected significant differences in bacterial composition between the control and PGR treatments; all PGR treatments enhanced the relative abundance of Proteobacteria and Acidobacteria, whose community structures could be used as a bacterial indicator for farmland (Kim et al., 2021). Proteobacteria and Acidobacteria were the dominant bacteria in the rhizosphere of kiwifruit as reported by Liu et al. (2020). The enhanced abundance of Proteobacteria and Acidobacteria may be explained by enhanced soil organic matter content observed in our study (Trivedi et al., 2013). Proteobacteria, taxonomically classified as copiotrophs, exhibit rapid growth under nutrient-sufficient conditions due to their utilization of labile carbon for metabolic activities and proliferation (Fierer et al., 2007; Trivedi et al., 2013). The phylum Acidobacteria is highly abundant in the rhizosphere and contributes to polysaccharides degradation and carbon cycling (Ward et al., 2009). While the maximum relative abundance for Gemmatimonadetes, Verrucomicrobia, and Chloroflexi was recorded in the control, similar findings were reported by Li et al. (2018), where more abundance of Verrucomicrobia was detected in melatonin-free replant soil. These results suggest that a high abundance of these bacteria may act negatively and hinder the process of decomposition and nutrient cycling ultimately reducing the growth of plants as evident from our biomass data, however, this needs to be elucidated further in detail.

At the genus level, the bacterial composition among different soil samples was the same, however, the proportion of each genus showed fluctuation (**Figure 6**, **Supplementary Table 1**). For instance, JG36-GS-52 was found only in MT and MT+14-HBR treatments. Lamia and Sphingobacteriaceae were detected in all soil treatments except SF and the control. In contrast to other soil treatments, MT and MT+14-HBR treatments showed the highest abundance of Proteobacteria, Myxococcales, Desulfarculaceae Bdellovibrio, Subgroup 22, Bryobacter, Rokubacteriales, and Anaerolineae, respectively. All PGR treatments significantly enhanced the abundance of Bradyrhizobium, Alphaproteobacteria, Dongia, bacterial-25, Acidobacteriaceae, and Gemmatirosa compared with the control (**Figure 6**). Bradyrhizobium was reported to be the predominant in the root nodules of mung bean (Quiza et al., 2015; Hakim et al., 2021). However, the detailed mechanism by which MT and MT+14-HBR enriched the rhizosphere bacterial communities demands further in-depth studies.

Mineral nutrients are absorbed by roots from the rhizosphere in an ionic state regulated by various transport proteins. Different macronutrients (N, P, K) and micronutrients (Zn, Cu, Mn, Fe) have been reported to play indispensable functions in different physiochemical processes occurring in plants (Maathuis and Diatloff, 2013; Victor Roch et al., 2019). Our results revealed that exogenous application of MT among alone treatments and MT+14-HBR in combined treatments significantly enhanced leaf and soil mineral nutrient contents as well as soil organic matter content (**Figures 5**, **7**). Our results were parallel to the previous study (Cheng et al., 2021), where the application of brassinosteroid enhanced growth via nutrient homeostasis in soybean seedlings under normal conditions. Similar findings have also been illustrated that enhanced soil organic matter content with manure and growth regulator (NAA) combination in tomatoes (Naz et al., 2018). Our results suggested that MT and MT+14-HBR regulated improvement in mineral nutrient and organic matter content might be the result of enhanced soil enzymatic and microbial activities contributing towards better nutrient availability in soil and leaf as evident from our soil enzymatic and microbial results (**Figures 4**–**6**). Previous studies have also proved the role of soil enzymes and beneficial microbes in nutrient cycling and retaining soil fertility status (Cui et al., 2016; Li et al., 2018).

In summary, our findings showed that the application of MT and MT+14-HBR significantly enhanced the growth parameters, biomass accumulation, photosynthetic pigments and antioxidant enzyme activity compared with the control. In addition, MT and MT+14-HBR treatments markedly increased the soil enzyme activities and soil mineral nutrient content, and improved nutrient absorption by plants. Furthermore, the highest abundance of bacterial communities at phylum and genus levels was recorded in MT and MT+14-HBR treated soils. The results suggested that MT and MT+14-HBR induced improvement in morphology and physiochemical attributes of kiwifruit seedlings were associated with significant enrichment of rhizosphere with beneficial bacterial communities and soil enzymes contributing towards enhanced nutrient cycling, degradation of organic matter, soil fertility, and ultimately improvement of plant growth.

## Data availability statement

The data presented in the study are deposited in the NCBI Sequence Read Archive Bioproject repository, accession number PRJNA842376. https://www.ncbi.nlm.nih.gov/sra/?term=PRJNA842367.

## Author contributions

XZ: Methodology, Validation, Writing – original draft. TH: Investigation, Methodology. YL: Investigation, Methodology. SH: Data curation, Investigation, Methodology, Writing – original draft. RP: Investigation, Methodology. TW: Investigation, Methodology. HD: Methodology, Validation. JW: Methodology, Resources. XL: Resources, Supervision. DL: Funding acquisition. HX: Funding acquisition, Supervision, Validation, Writing – review & editing.
