# Supplementary material for: Melatonin and 14-hydroxyed brassinosteroid combined promote kiwifruit seedling growth by improving soil microbial distribution, enzyme activity and nutrients uptake
Source: Front Plant Sci. 2024 Feb 8;15:1336116. doi: 10.3389/fpls.2024.1336116 (PMC10881855; doi:10.3389/fpls.2024.1336116)
Supplement: Supplementary file 1 [file DataSheet_1.docx]

**Table S1** Application of primers and sequences

| Primer Name | Purpose | Sequence (5’-3’) |
| --- | --- | --- |
| *qSOD*  *qSOD[Cu-Zn]*  *qPOD1*  *qPOD2*  *qCAT1*  *qCAT6*  *qActin* | qPCR | F: AAAGGCGGGCTAGGGTTAGG  R: TGGAAGATCCGGGAGCGATAF:  F: GCGGGTGACCTGGGAAACAT  R: AGGCTCTGCCGACGACTGAAR:  F: CTGCCCAGCACTAGACACAA  R: GTCCTGGTCGGACGTAAAAAF:  F: CCGAACGCGGTCCAGTATGT  R: TTGGTCCTCTTGTCGGTGGC  F: ACCTGAGTGCCCTTTAAGCC  R: TTTGGGTATGAACGAGTTGG  F: CTACATCCGCATCACCTTCG  R: TATCAGATTCGCTCCCGTCA  F: TGCATGAGCGATCAAGTTTCA  R: TGTCCCATGTCTGGTTGATGA |

**Table S2** Statistics of sample size and alpha diversities.

|  | Observed_OTUs | Shannon | Simpson | Chao1 | Coverage |
| --- | --- | --- | --- | --- | --- |
| CK | 3012.33 | 10.78 | 0.9989 | 3406.53 | 0.962 |
| MT | 2900.67 | 10.77 | 0.9990 | 3213.72 | 0.967 |
| SF | 2931.33 | 10.74 | 0.9989 | 3269.86 | 0.965 |
| HBR | 2697.67 | 10.62 | 0.9988 | 3000.08 | 0.970 |
| SF+MT | 2784.33 | 10.72 | 0.9990 | 3091.01 | 0.969 |
| MT+HBR | 2855.00 | 10.73 | 0.9990 | 3210.08 | 0.966 |

**Table S3** Principal component analysis (PCA) comparison of bacterial communities between control and PGRs treated soil samples, at genus level.

| **Bacterial group** | **CK** | **MT** | **SF** | **14-HBR** | **SF+MT** | **MT+14-HBR** |
| --- | --- | --- | --- | --- | --- | --- |
| Sphingomonas | 2.90 | 2.02 | 3.22 | 2.46 | 2.19 | 1.88 |
| KF-JG30-C25 unclassified | 1.56 | 1.28 | 1.22 | 1.45 | 0.86 | 1.79 |
| Hyphomicrobiaceae_unclassified | 0.36 | 0.49 | 0.66 | 0.39 | 0.52 | 0.41 |
| SC-I-84_unclassified | 0.92 | 2.26 | 2.36 | 2.09 | 2.51 | 2.24 |
| Rhodanobacter | 0.56 | 0.39 | 0.70 | 0.63 | 0.71 | 1.63 |
| Burkholderia-Caballeronia | 0.50 | 2.16 | 0.63 | 0.76 | 0.72 | 0.64 |
| Micropepsaceae_unclassified | 1.18 | 1.23 | 2.21 | 1.49 | 1.61 | 1.43 |
| Cupriavidus | 0.11 | 0.21 | 0.22 | 0.13 | 0.25 | 0.19 |
| Janthinobacterium | 0.01 | 0.08 | 0.03 | 0.00 | 0.00 | 0.00 |
| Burkholderiales_unclassified | 0.29 | 0.43 | 0.45 | 0.45 | 0.52 | 0.48 |
| Betaproteobacteria_unclassified | 0.31 | 0.32 | 0.31 | 0.39 | 0.35 | 0.42 |
| Xylophilus | 0.06 | 0.05 | 0.11 | 0.08 | 0.10 | 0.05 |
| Dokdonella | 0.27 | 0.32 | 0.34 | 0.44 | 0.42 | 0.44 |
| JG36-GS-52_unclassified | 0.00 | 0.01 | 0.00 | 0.00 | 0.00 | 0.01 |
| Pseudolabrys | 0.42 | 0.32 | 0.43 | 0.32 | 0.34 | 0.28 |
| Bradyrhizobiaceae_unclassified | 0.09 | 0.09 | 0.11 | 0.10 | 0.11 | 0.06 |
| Mesorhizobium | 0.15 | 0.18 | 0.21 | 0.24 | 0.23 | 0.21 |
| Proteobacteria_unclassified | 0.68 | 0.75 | 0.66 | 0.71 | 0.72 | 0.83 |
| KF-JG30-B3_unclassified | 0.24 | 0.33 | 0.35 | 0.33 | 0.32 | 0.33 |
| Rhodospirillaceae_unclassified | 0.25 | 0.25 | 0.31 | 0.25 | 0.28 | 0.29 |
| Reyranella | 0.38 | 0.36 | 0.40 | 0.43 | 0.47 | 0.40 |
| Haliangium | 1.46 | 1.01 | 1.03 | 1.18 | 1.33 | 1.40 |
| Myxococcales_unclassified | 0.25 | 0.32 | 0.20 | 0.27 | 0.20 | 0.29 |
| Deltaproteobacteria_unclassified | 0.28 | 0.31 | 0.09 | 0.30 | 0.37 | 0.29 |
| Desulfarculaceae_unclassified | 0.52 | 0.81 | 0.53 | 0.62 | 0.66 | 0.78 |
| Ellin6067 | 0.71 | 0.79 | 0.89 | 0.82 | 0.90 | 0.87 |
| TRA3-20_unclassified | 0.62 | 0.54 | 0.70 | 0.50 | 0.76 | 0.62 |
| mle1-7 | 0.22 | 0.70 | 0.63 | 0.63 | 0.82 | 0.77 |
| Nitrosospira | 0.07 | 0.13 | 0.24 | 0.20 | 0.25 | 0.15 |
| IS-44 | 0.11 | 0.08 | 0.03 | 0.10 | 0.08 | 0.10 |
| A21b_unclassified | 0.22 | 0.55 | 0.33 | 0.46 | 0.65 | 0.60 |
| MND1 | 1.69 | 1.39 | 1.17 | 1.27 | 1.56 | 1.70 |
| Gammaproteobacteria_unclassified | 0.27 | 0.24 | 0.20 | 0.30 | 0.28 | 0.31 |
| Rhodanobacteraceae_unclassified | 0.11 | 0.12 | 0.12 | 0.16 | 0.17 | 0.20 |
| Xanthomonadaceae_unclassified | 0.37 | 0.36 | 0.39 | 0.40 | 0.47 | 0.43 |
| Lysobacter | 0.24 | 0.26 | 0.17 | 0.37 | 0.35 | 0.31 |
| Dyella | 0.18 | 0.17 | 0.13 | 0.26 | 0.35 | 0.33 |
| Chujaibacter | 0.17 | 0.20 | 0.23 | 0.30 | 0.25 | 0.40 |
| Polycyclovorans | 0.52 | 0.25 | 0.04 | 0.46 | 0.33 | 0.55 |
| Xanthobacteraceae_unclassified | 0.37 | 0.49 | 0.58 | 0.45 | 0.49 | 0.44 |
| Bradyrhizobium | 0.29 | 0.45 | 0.44 | 0.51 | 0.51 | 0.42 |
| Novosphingobium | 0.15 | 0.16 | 0.17 | 0.16 | 0.11 | 0.15 |
| Alphaproteobacteria_unclassified | 1.42 | 1.52 | 1.79 | 1.66 | 1.77 | 1.71 |
| Elsterales_unclassified | 1.35 | 1.22 | 1.05 | 1.22 | 1.00 | 1.11 |
| Dongia | 0.23 | 0.29 | 0.37 | 0.34 | 0.41 | 0.31 |
| Amb-16S-1034_unclassified | 0.03 | 0.02 | 0.01 | 0.03 | 0.01 | 0.03 |
| P3OB-42_unclassified | 0.07 | 0.07 | 0.07 | 0.06 | 0.07 | 0.10 |
| bacteriap25_unclassified | 0.26 | 0.49 | 0.38 | 0.40 | 0.49 | 0.45 |
| Bdellovibrio | 0.04 | 0.07 | 0.02 | 0.05 | 0.06 | 0.08 |
| Subgroup_2_unclassified | 4.21 | 3.44 | 4.14 | 3.74 | 3.16 | 3.56 |
| Subgroup_22_unclassified | 0.34 | 0.41 | 0.20 | 0.27 | 0.36 | 0.40 |
| Terracidiphilus | 0.20 | 0.18 | 0.33 | 0.22 | 0.22 | 0.27 |
| Edaphobacter | 0.06 | 0.08 | 0.08 | 0.09 | 0.09 | 0.10 |
| Subgroup_5_unclassified | 0.43 | 0.48 | 0.30 | 0.38 | 0.48 | 0.49 |
| Subgroup_6_unclassified | 3.93 | 4.18 | 3.40 | 3.96 | 4.15 | 4.12 |
| Paludibaculum | 0.01 | 0.02 | 0.02 | 0.01 | 0.03 | 0.04 |
| Subgroup_13_unclassified | 0.78 | 0.79 | 0.71 | 0.86 | 0.77 | 0.83 |
| JGI_0001001-H03 | 0.22 | 0.18 | 0.10 | 0.24 | 0.19 | 0.18 |
| 11-24_unclassified | 0.18 | 0.18 | 0.08 | 0.23 | 0.38 | 0.34 |
| Holophaga | 0.46 | 0.26 | 0.31 | 0.36 | 0.41 | 0.40 |
| Subgroup_7_unclassified | 0.88 | 1.35 | 1.05 | 1.29 | 1.52 | 1.62 |
| Acidobacteriaceae_unclassified | 0.30 | 0.37 | 0.54 | 0.32 | 0.36 | 0.37 |
| Acidobacteriales_unclassified | 2.34 | 3.31 | 2.73 | 2.80 | 3.05 | 3.04 |
| Subgroup_11_unclassified | 0.08 | 0.08 | 0.07 | 0.12 | 0.14 | 0.13 |
| Candidatus_Solibacter | 1.88 | 1.79 | 2.04 | 1.99 | 2.14 | 2.32 |
| Bryobacter  Blastocatellaceae_unclassified | 1.80  0.20 | 2.34  0.19 | 1.84  0.22 | 1.91  0.31 | 2.03  0.28 | 2.15  0.30 |
| RB41 | 1.06 | 1.43 | 1.03 | 1.25 | 1.83 | 2.05 |
| Rokubacteriales_unclassified | 1.28 | 2.32 | 1.73 | 1.49 | 2.00 | 2.64 |
| KD4-96_unclassified | 1.28 | 0.90 | 1.02 | 0.94 | 0.80 | 0.87 |
| Anaerolineae_unclassified | 0.08 | 0.09 | 0.04 | 0.07 | 0.06 | 0.09 |
| Iamia | 0.00 | 0.02 | 0.00 | 0.01 | 0.01 | 0.01 |
| Actinobacteria_unclassified | 0.62 | 0.49 | 0.55 | 0.79 | 0.64 | 0.75 |
| Rubrobacteria_unclassified | 0.08 | 0.10 | 0.09 | 0.10 | 0.08 | 0.06 |
| Gaiellales_unclassified | 0.62 | 0.58 | 0.64 | 0.57 | 0.52 | 0.64 |
| Gemmatirosa | 0.30 | 0.36 | 0.45 | 0.37 | 0.40 | 0.44 |
| Gemmatimonas | 2.33 | 1.65 | 1.81 | 2.00 | 2.05 | 2.39 |
| Gemmatimonadetes_unclassified | 0.69 | 0.64 | 0.61 | 0.67 | 0.64 | 0.72 |
| Gemmatimonadaceae_unclassified | 4.04 | 2.63 | 3.91 | 2.99 | 3.42 | 3.20 |
| BD2-11_terrestrial_group_unclassified | 0.09 | 0.05 | 0.13 | 0.06 | 0.08 | 0.06 |
| Planctomycetales_unclassified | 0.55 | 0.66 | 0.47 | 0.43 | 0.29 | 0.26 |
| Pirellulaceae_unclassified | 0.43 | 0.58 | 0.35 | 0.36 | 0.19 | 0.16 |
| Pirellula | 0.50 | 0.64 | 0.39 | 0.41 | 0.21 | 0.19 |
| Gemmataceae_unclassified | 0.85 | 0.79 | 0.48 | 0.37 | 0.37 | 0.44 |
| WD2101_soil_group_unclassified | 2.38 | 2.90 | 2.68 | 1.82 | 1.81 | 1.91 |
| OM190_unclassified | 0.46 | 0.50 | 0.15 | 0.27 | 0.34 | 0.37 |
| Verrucomicrobium | 0.01 | 0.01 | 0.00 | 0.01 | 0.00 | 0.00 |
| Prosthecobacter | 0.01 | 0.01 | 0.00 | 0.01 | 0.02 | 0.02 |
| Luteolibacter | 0.07 | 0.04 | 0.00 | 0.08 | 0.03 | 0.03 |
| Opitutus | 0.19 | 0.19 | 0.08 | 0.16 | 0.07 | 0.10 |
| Sphingobacteriaceae_unclassified | 0.00 | 0.01 | 0.00 | 0.002 | 0.01 | 0.04 |
| Candidatus_Adlerbacteria_unclassified | \| 0.37 \| \| --- \| | 0.42 | 0.18 | 0.21 | 0.13 | 0.12 |
| Saccharimonadales unclassified | 1.02 | 1.11 | 1.05 | 1.10 | 0.95 | 1.09 |


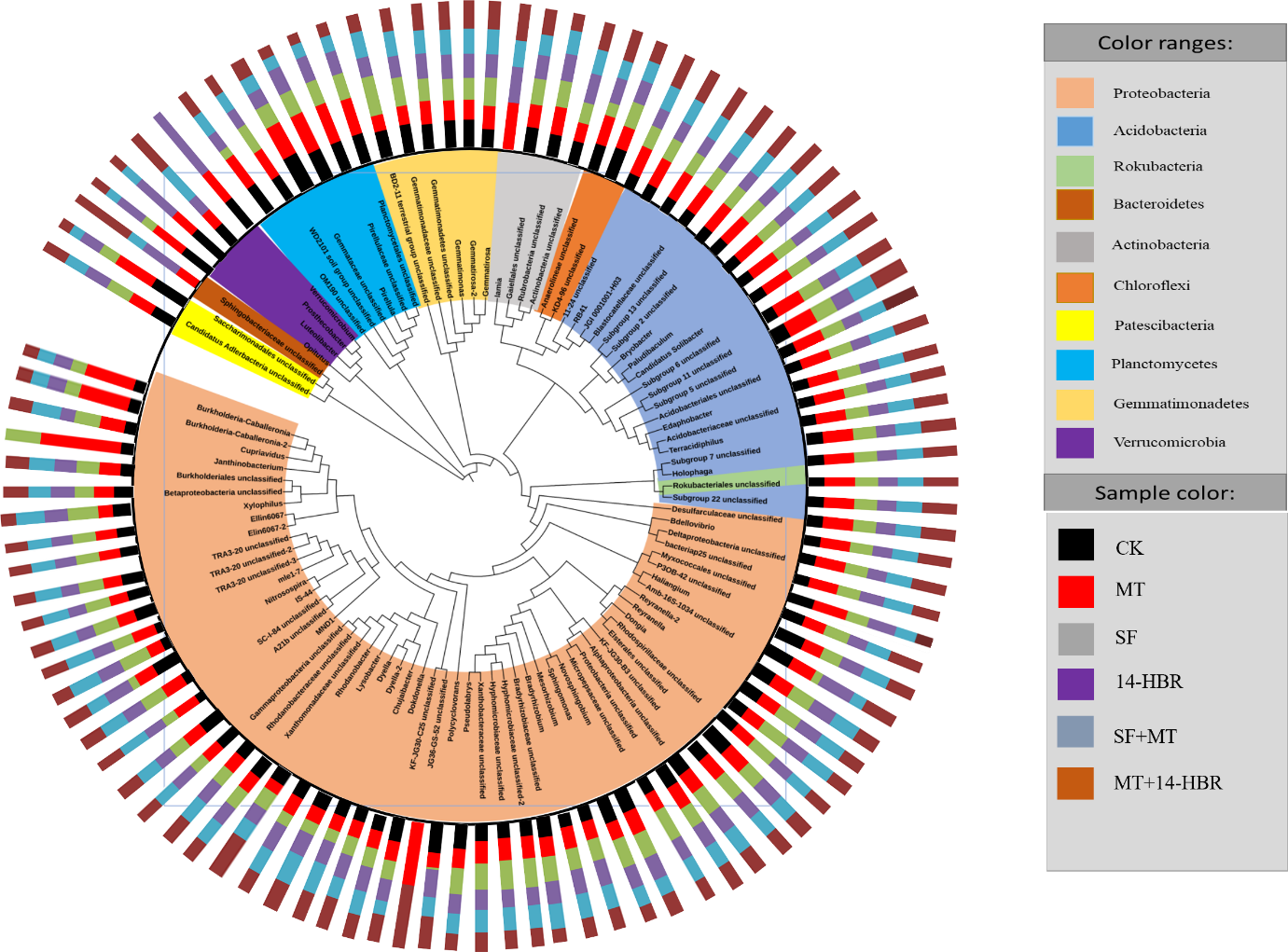


**Figure S1** Comparison of bacterial communities between control and PGRs treated soil samples at genus level.
